# COVID‐19 and Policy Responses by International Organizations: Crisis of Liberal International Order or Window of Opportunity?

**DOI:** 10.1111/1758-5899.12975

**Published:** 2021-06-23

**Authors:** Maria Josepha Debre, Hylke Dijkstra

**Affiliations:** ^1^ University of Potsdam; ^2^ Maastricht University

## Abstract

The liberal international order is being challenged and international organizations (IOs) are a main target of contestation. COVID‐19 seems to exacerbate the situation with many states pursuing domestic strategies at the expense of multilateral cooperation. At the same time, IOs have traditionally benefited from cross‐border crises. This article analyzes the policy responses of IOs to the exogenous COVID‐19 shock by asking why some IOs use this crisis as an opportunity to expand their scope and policy instruments? It provides a cross‐sectional analysis using original data on the responses of 75 IOs to COVID‐19 during the first wave between March and June 2020. It finds that the bureaucratic capacity of IOs is significant when it comes to using the crisis as an opportunity. It also finds some evidence that the number of COVID‐19 cases among the member states affects policy responses and that general purpose IOs have benefited more.

## Abstract 2

Cross‐border crises such as COVID‐19 can present IOs with windows of opportunity to expand their scope and develop new policy instruments.

**Policy Implications**

- International organizations have responded very differently to COVID‐19. Evidence from 75 international organizations shows that those with broad policy objectives have further expanded their scope and instruments. International organizations with a narrow focus have stuck to existing instruments.
- Bureaucratic capacity explains the ability of international organizations to use crises as an opportunity for institutional development. It is imperative that member states further invest in the bureaucratic capacity of international organizations, particularly in professional staff, to handle future crises.
- When considering future reforms of international organizations, member states should focus on strengthening the competences of the executive bodies to initiate policy to help organizations work better during crises.
- As we exit the pandemic it is tempting to concentrate on domestic affairs, including revisiting healthcare and pandemic response systems, but policy makers are advised to further develop global governance in these areas as well. While COVID‐19 was initially seen as a major challenge to the liberal international order, it may well result in a deepening of global governance in the longer term.

## 1 At a critical juncture

The liberal international order is in crisis and international organizations (IOs) are a main target of contestation.^1^ Increasing politicization in combination with member states cutting resources, withdrawing from institutions, or setting up alternative venues for cooperation has impacted IOs in profound ways (e.g. von Borzykowski and Vabulas, 2019; Hale et al., 2013; Patz and Goetz, 2019; Zürn, 2018). The exogenous shock of COVID‐19 has further exacerbated pressures with many states pursuing domestic strategies at the expense of multilateral cooperation. Academics and pundits have therefore been quick to characterize COVID‐19 as yet another blow to the liberal international order (e.g. Kahl and Wright, 2021; Kenwick and Simmons, 2020; Mahbubani, 2020; Norrlöf, 2020a, 2020b). At the same time, we know that IOs are often created exactly to address cross‐border problems (e.g. Rittberger et al., 2019) and that they regularly benefit from crises (Kreuder‐Sonnen, 2019; Monnet, 1978; Olsson and Verbeek, 2018; Schimmelfennig, 2018).

This article analyzes the policy responses of 75 major IOs in the context of the exogenous COVID‐19 shock by asking why some IOs use this crisis as an opportunity to expand their scope and policy instruments, whereas other IOs use existing instruments or barely engage with COVID‐19 at all? The purpose is not to assess the effectiveness of IOs, but rather to examine whether IOs proactively used this exogenous shock as an opportunity to expand their activities during the first wave between March and June 2020. Even though the COVID‐19 crisis continues to date, we assume that the initial responses of IOs were essential because critical junctures are brief moments in time and future developments with regard to legalization of policy expansions are likely path dependent on these initial responses (Gerschewski, 2021; Pierson, 2000). There are also first‐mover advantages that affect how global governance gets reordered.

In line with research on international public administration, this article shows that the bureaucratic capacity of IOs is significant when it comes to providing continuity of operations and using the crisis as an opportunity: IOs with large secretariats are more likely to expand their scope and policy instruments in response to COVID‐19. Such IOs may be able to reassign staff to work on crisis response, are more likely to have relevant in‐house expertise, and can put forward policy proposals. We also find some evidence that IOs with delegated agenda‐setting authority are better at using the crisis as an opportunity. While the operations of most IOs are affected by COVID‐19, we also control for differences between IOs with regard their likelihood to respond to the crisis. We find, in this respect, that general purpose IOs are more likely to expand their scope and instruments compared to task‐specific IOs (cf. Hooghe et al., 2019). We also find some evidence that the number of COVID‐19 cases among IO members helps to explain IO responses. We do not find, however, that IOs with mandates in heavily affected policy fields such as health, trade, and border management are more likely to expand.

The article first conceptualizes policy responses of IOs and maps how 75 major IOs varied in trying to expand their scope and policy instruments during the initial crisis months from March until the end of the study period in June 2020. It then derives two sets of institutional hypotheses to explain observed variation in policy responses, discusses research design, and operationalizes the variables. The empirical section provides an ordered logit model that tests all hypotheses and shows the significance of large secretariats in explaining the expansion of scope and policy instruments. The article concludes by reflecting on the consequences of these findings for the state of the liberal international order.

## 2 Policy responses by IOs during COVID‐19

Crises are commonly considered important moments in organizational history because they offer opportunities to change organizational processes (Boin et al., 2016). Punctuated equilibrium theory (PET), in particular, focuses on institutional change as a result of an exogenous shock with a short‐term time horizon (Gerschewski, 2021). This makes COVID‐19 an excellent test case, since it is clearly exogenous and required an immediate response by many IOs. COVID‐19 will, of course, also have longer‐term consequences. Yet in the short‐term, exogenous shocks have the potential to set institutions on a new track, or can also simply represent opportunities to change direction and branch out from an original path (Gerschewski, 2021; see also on PET: Baumgartner and Jones, 1993, Colgan et al., 2012; Lundgren et al., 2018). Thus, choices made by IOs, during the brief initial window after COVID‐19 was declared a pandemic in March 2020, can induce self‐reinforcing processes and canalize future developments down a new path. IOs may also benefit from first‐mover advantages in a crowed global governance landscape. Conversely, if IOs did not act during the first wave, they have little to build on to induce a long‐term process of institutional change.

We are thus interested in institutional change as a result of COVID‐19 and not policy effectiveness. We understand therefore the concept of policy response as any measure taken up by an IO bureaucracy to deal with the pandemic. We are particularly interested whether this has resulted in proposals for an expanded *scope of action* and/or new *policy instruments* (Hooghe et al., 2019; Koremenos et al., 2001, 2001). Thus, policy responses encompass both the tasks performed by the institution, including in new policy areas, as well as employed instruments such as funding mechanisms, coordination tools, databases, research lines, or training programs. Our concept does not, however, cover policies that IOs have taken up to manage their internal operations such as regulations on remote work.

To give an example, the European Commission has negotiated collective agreements with pharmaceutical companies on vaccines. The European Union has furthermore agreed to a 100bln euro temporary unemployment scheme. It has also made available existing policy instruments such as the European Stability Mechanism for health purposes and has agreed to a new 750bln euro recovery fund financed through joint debt. In other words, the EU has clearly increased its *policy scope* into the areas of health and social policy while using existing and newly developed *policy instruments* (see Brooks and Geyer, 2020; Ladi and Tsarouhas, 2020; Wolff and Ladi, 2020). The World Trade Organization (WTO), on the other hand, only offered some updates on trade statistics in response to the pandemic. Even though it remains the focal institution for world trade, which was heavily affected by COVID‐19, it thus did not expand its *policy scope* nor did it develop new *policy instruments*.

IO responses during the first wave of the COVID‐19 crisis can be divided into three categories. First, some IOs essentially shut down or merely issued declaratory statements without really responding to COVID‐19. Second, some IOs managed to perform their established tasks with existing instruments. Third, some IOs were able to take on new tasks and/or initiative new policy instruments, as the example of the EU shows (see Table 1 for coding examples). We code policy responses on a six‐point scale, with IOs that have not responded at all to COVID‐19 coded as zero and IOs that have only issued a discursive response as low (1). IOs that are fulfilling their established tasks or/and use established policy instruments are coded as low‐medium (2) and medium (3), and as high (4) and very high (5) if they are taking on new tasks or/and have initiated new policy instruments (see Table A and B, Appendix for full coding of all IOs; we also perform a robustness check with a three‐point scale: low, medium, high, see Table C, Appendix).

**Table 1:** Examples of coding for six IOs by policy instruments and scope

IO name	Policy instruments	Coding	Policy scope	Coding	Scale
European Union (EU)	‐ Temporary unemployment scheme ‐ Recovery fund financed through joint debt ‐ European Stability Mechanism for health purposes	*New policy instruments*	‐ Health ‐ Social policy ‐ Border management ‐ Finance	*Scope expansion*	Very high (= 5)
International Monetary Fund (IMF)	‐ Initiation of new short‐term liquidity lines ‐ Repurposing of existing funds for emergency relief	*New policy instruments*	‐ Trade ‐ Finance	*Existing scope*	High (= 4)
Southern African Development Community (SADC)	‐ Harmonization of Health Guidelines ‐ Coordination of trading rules for cross‐border transports ‐ Coordination with other global and regional IOs ‐ Negotiations with India on preferential trading in medical supplies	*Existing policy instruments*	‐ Health ‐ Trade ‐ Political coordination	*Existing scope*	Medium (= 3)
World Trade Organization (WTO)	‐ Joint statement with IMF ‐ Limited reporting on international trade flows	*Limited use of existing policy instruments*	Trade (Monitoring)	*Existing scope*	Low‐Medium (= 2)
Central African Economic and Monetary Union (CEMAC)	Statement by Secretary General	*Discursive response*	No activity reported	*Not applicable*	Low (= 1)
International Whaling Commission (IWhale)	No response	*No response*	No activity reported	*Not applicable*	No response (= 0)

We have coded policy responses for all IOs included in the *Measuring International Authority* (MIA) dataset by Hooghe et al. (2017). These are the 78 politically most relevant IOs since 1950.^2^ To code policy responses, we have consulted the websites of all IOs to gather information on their activities since the start of the pandemic in March 2020 until the end of the study period in June 2020. We observe that most IO responses were formulated during April and early May and consider that after June 2020 the initial window of opportunity had passed. Many IOs obviously remain active in dealing with COVID‐19, but the critical juncture which could set IOs on new paths is over. Relying on online data gathering might not cover all daily practices. Nevertheless, it helps to capture a snapshot of IO capacity to communicate actions online, and thus also serves as a proxy to measure how well IOs are able to keep up policy functions.

**Figure 1:**
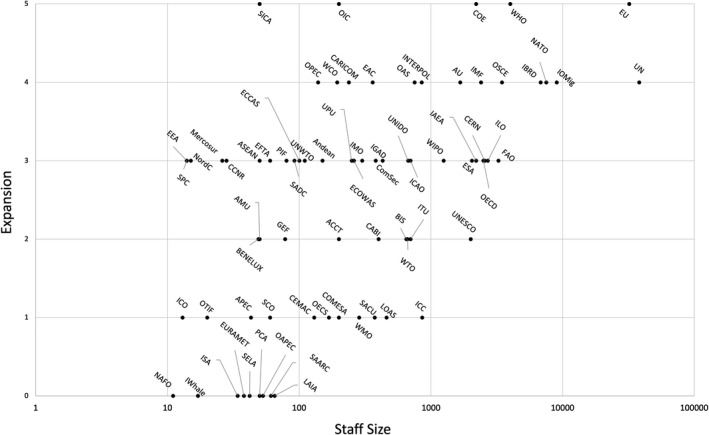
Variation in scope and policy instrument expansion by staff size.

We find that 22 IOs did not respond to COVID‐19 at all during the first wave or only issued statements about the importance of the pandemic for their policy field (coded 0 or 1) (see Figure 1). While many of these are task‐specific IOs, which we would not necessarily expect to be heavily affected by COVID‐19 (e.g. the International Whaling Commission), there are several economic and trading IOs (e.g. International Coffee Board) as well as regional organizations (e.g. CEMAC) that are dealing with policy fields directly hit by COVID‐19.

The majority of IOs (35) used their existing policy instruments and/or stayed within their existing scope to deal with the challenges (coded 2 or 3). They collected data, offered webinars, or coordinated between member states. An example is the Universal Postal Union (UPU), which had to deal with the fact that international postal services were heavily disrupted. In May 2020, only one‐in‐two postal items reached their international destination due to the cancellation of passenger flights (Universal Postal Union, 2020). The response of the UPU largely focused on providing analysis and expertise, sharing best practices, and initiating small projects such as delivering personal protective equipment to United Nations (UN) field operations.

Finally, we found 18 IOs to venture into new tasks or initiate new policy instruments (coded 4 or 5). The Organization for Security and Co‐operation in Europe (OSCE) started to provide humanitarian aid. The International Monetary Fund (IMF) initiated and repurposed a number of funding instruments to support least developed countries and provided short‐term liquidity lines. Some IOs even used the crisis to propose some potentially far‐reaching changes: the EU agreed to joint debt, the WHO has taken up global supply change management in coordination with the World Food Programme (WFP), and the Council of Europe (CoE) ventured into health policy by financing liquidity shortages. The CoE also used the COVID‐19 crisis to reposition itself, through a public relations offensive, as the focal institution for human rights in Europe, with a number of initiatives surrounding the organization and legality of cyber justice.

IOs have responded differently to COVID‐19. Some have barely engaged at all, whereas others have seen an opportunity in this crisis. The remainder of the article seeks to explain such variation. It is, in this respect, important to reiterate that our dependent variable should not be equated with effectiveness of response. Some IOs have actually been heavily contested for their slow or inadequate initial response. The EU and WHO are perhaps the most prominent examples of IOs of which many people expected more (Johnson, 2020). An initial lack of effectiveness may also drive IOs into new territory. As previous health crises show, the WHO ended up with expanded authority in the wake of the SARS and Ebola crisis exactly as a result of identified shortcomings (Kreuder‐Sonnen, 2019). A demand for increased authority of IOs may also result in contestation. EU member states, for instance, fought heavily over the allocation of funds and the issue of joint debts, even if the EU eventually came out with innovative funding instruments, a larger budget, and an increased policy scope (Wolff and Ladi, 2020).

## 3 COVID‐19, institutional design, and policy responses

This article argues that variation in institutional design of IOs conditions the likelihood to be able to respond and adapt to unforeseen crises such as COVID‐19 (Hooghe et al., 2019; Koremenos et al., 2001). This section puts forward two sets of hypotheses on the *authority* and the *bureaucratic capacity* of IOs. It posits that IOs with more authority and bureaucratic capacity are better able to respond to exogenous shocks and have the ability to strategically use the crisis to their advantage.

As already noted in the introduction, much of the initial commentary has pointed out how COVID‐19 further undermines the liberal international order (Greitens, 2020; Kenwick and Simmons, 2020). Various observers noted that COVID‐19 is the first international crisis since the end of the Cold War where the United States does not play a coordinating role (Norrlöf, 2020). Kahl and Wright (2021, title) talk about the ‘end of the old international order.’ China seems to accelerate its development as a great power by embarking on ‘mask diplomacy’ and tightening its hold on Hong Kong (Mahbubani, 2020). Such geopolitics also caused gridlock within IOs with notably the WHO losing funding from the United States and being accused of China‐bias. Many states furthermore pursued domestic strategies, such as closing borders, with nationalization coming at the expense of multilateral cooperation (Bouckaert et al., 2020; Ferhani and Rushton, 2020; Johnson, 2020).

While COVID‐19 presents a challenge to the liberal international order, we also know that cross‐border problems (with COVID‐19 as a case in point) often provide impetus for (renewed) cooperation (Rittberger et al., 2019). Indeed, the WHO and its predecessors were originally established precisely because ‘diseases do not stop at borders’. It is also well‐known that IOs regularly benefit from crises, because established orders become fluid when the stakes are high and rapid response is imperative (Kreuder‐Sonnen, 2019; Olsson and Verbeek, 2018; Schimmelfennig, 2018; Stone, 2011). Crises also often highlight the previous shortcomings of IOs resulting in more delegation of authority (Jones et al., 2016). As one of the founding fathers of the EU famously said, ‘Europe will be forged in crises, and will be the sum of the solutions adopted for those crises’ (Monnet, 1978, p. 417). From this perspective, it is not surprising that some IOs have used COVID‐19 to expand their scope and policy instruments.

To explain the ability of IOs to benefit from exogenous shocks in the short‐term requires us to pay attention to institutional design. The institutions of IOs, of course, evolve over time. Yet in the short‐term, they condition the likelihood of IOs to be able to respond and adapt to unforeseen circumstances and crises. When COVID‐19 hit, IOs had to craft responses in line with the institutions they had at their disposal. While there are potentially many relevant institutional design features, the two key questions are whether IO had sufficient *authority* to formulate policy responses, including those expanding the scope of cooperation and proposing new policy instruments, and the *bureaucratic capacity* to do so in a strategic and credible manner.

The authority of IOs is important in times of crisis because it determines where and how policy responses and decisions are made. This institutional setting, in turn, provides the opportunity structure which affects whether IOs can cope with or even benefit from COVID‐19 (van Hecke et al., 2021). Authority consists of two dimensions: the delegation and pooling of sovereignty (Hooghe et al., 2017). Delegation involves ‘a conditional grant of authority by member states to an independent body’ (Hooghe et al., 2017, p. 21), such as a secretariat which may set the decision‐making agenda by proposing policies or take day‐to‐day decisions. It is likely that IOs with high degrees of delegation will be better able to respond to crises. First, if secretariats have some decision authority, they can make emergency decisions in the interest of the continued operations of the IO even if member states in the executive organs have difficulty reaching consensus. Second, if secretariats have agenda‐shaping powers they can proactively propose responses and new policy initiatives.

Pooling concerns ‘joint decision making among the principals themselves’ (Hooghe and Marks, 2015, p. 307) and involves, amongst others, the decision‐rule and whether member states have vetoes, particularly in the executive organs. Other things being equal, majority‐voting not only helps to speed up decision‐making, which is critical in crises, it also avoids gridlock due to opposing veto‐players (Hale et al., 2013; Tsebelis, 2002). If IOs have high degrees of authority, either through the delegation or pooling of sovereignty, they are more likely to be in position to cope with crises or actually use crises as opportunities to adapt policies. Since there is a trade‐off between the pooling and delegation of sovereignty (Hooghe and Marks, 2015), as IOs with a large membership typically pool rather than delegate sovereignty, authority is tested through two separate hypotheses:

*H1a*: IOs with higher delegated sovereignty will more likely expand their scope and policy instruments during crises.

*H1b*: IOs with higher pooled sovereignty will more likely expand their scope and policy instruments during crises.

IOs should also have the bureaucratic capacity to respond to crises (Bauer and Ege, 2016; Heldt and Schmidtke, 2017). With COVID‐19, meetings were cancelled, and officials have been working from home. It is therefore not guaranteed that IOs have the ability to formulate adequate policy responses. In line with advances on international public administration (e.g. Bauer et al., 2017; Knill and Bauer, 2016), this article tests three aspects of bureaucratic capacity: staff, budget, and leadership.

First, the presence of a substantial secretariat will more likely allow IOs to benefit from crises. IOs with substantial staff resources may be able to reassign staff members to work on crisis response. They may also have strong in‐house expertise which they can use to pursue organizational interests (Barnett and Finnemore, 2004; Bauer and Ege, 2016; Eckhard and Ege, 2016; Hawkins et al., 2006). While IOs with large bureaucracies may experience institutional pathology and not be flexible enough to grasp opportunities (Barnett and Finnemore, 2004), Gray (2018) finds that quality staff is critical for the vitality of IOs and Johnson (2014) shows how secretariat staff act opportunistically when designing new institutions. While some IOs – such as the EU, UN, and WHO – have substantial secretariat resources, many of the 75 major IOs have in fact rather limited staffs often below 50 officials (Debre and Dijkstra, 2021). If there are very few actual policy officers, they may simply be overwhelmed with guaranteeing the continuation of operations and may not have the ability to proactively propose new policy instruments.

Second, available budget is also important. IOs vary significantly with regard to available funding, with some regulatory IOs (e.g. World Trade Organization) mostly having administrative budgets and operational IOs (e.g. World Bank) having sizeable funds (Rittberger et al., 2019). Due to institutional rules of IOs and lengthy budgetary cycles (Patz and Goetz, 2019), it is much more likely that IOs which already have substantial funds and experience in disbursing them will be treated as focal institutions in COVID‐19 response (see also Ege and Bauer, 2017; Goetz and Patz, 2017). They might free up funds, use flexibility rules to reallocate funds across policies, or set up new funding instruments. In light of the available data (see further below), we limit the analysis to overall budgets of IOs. While a more elaborate conceptualization of budgetary politics in crises is worthwhile for further studies, it is also reasonable that IOs which do not have substantial budgets are not likely to get them, in the short‐term, simply for the purpose of responding to COVID‐19.

Finally, it is important to pay attention to leadership which is critical in times of crises (e.g. Boin et al., 2016). While there are various determinants of public leadership, it seems that seniority and experience are particularly relevant for IOs in times of crises (Kille and Scully, 2003; Young, 1991). It is more likely that more senior and experienced politicians will identify the opportunities in crises rather than solely focus on continuity of operations. Officials with experience at the highest level may also be more used to working across organizational boundaries and overcoming formal and institutional constraints (Hall and Woods, 2018). Finally, senior politicians are more likely to have serious international networks and close connections to national capitals.

This leads to the following set of hypotheses:

*H2a*: IOs with a larger secretariat will more likely expand their scope and policy instruments of IOs during crises.

*H2b*: IOs with a larger budget will more likely expand their scope and policy instruments during crises.

*H2c*: IOs with a senior leader at the helm of the secretariat will more likely expand their scope and policy instruments during crises.

## 4 Research design

This article tests these institutional hypotheses through an ordered logistic regression analysis of policy responses by 75 major IOs. The dependent variable has already been conceptualized and operationalized above and is measured on a six‐point scale. This section discusses the independent and control variables.

To test hypothesis 1a and 1b, we use the MIA dataset by Hooghe et al. (2017) on delegation and pooling. Their extensive dataset includes aggregate measures of both concepts as well as scores for a number of sub‐dimensions. As a starting point, we include the two aggregated variables to measure the degree of decision‐making authority conferred to IO bodies (*delegation*) and the degree of joint decision‐making in the collective IO body (*pooling*). We use the values for the last available year in the dataset (2010). Since pooling and delegation are relatively stable measures and have not drastically changed between 2005 and 2010 (Hooghe et al., 2019), we are confident that the values remain valid representations of IO authority in 2020.

In addition to both aggregate measures of *delegation* and *pooling*, we include four variables from Hooghe et al. (2017) on sub‐dimensions: *delbudget* and *delpolicy* measure the extent to which budgetary allocation and agenda‐setting powers have been delegated to the secretariat or executive bodies. In case of delegated agenda‐setting powers, IOs may be able to suggest new initiatives, whereas budgetary powers should allow for more discretion in allocating funding to COVID‐19 responses. For pooling, we similarly include the sub‐dimensions of *poolbudget* and *poolpolicy*, which measure the degree to which decision‐making is jointly exercised with regard to policy‐making and budgetary matters. Majority‐voting in IOs should, in this respect, allow more easily for scope expansion and new policy instruments.

We test hypotheses 2a–c on the basis of the readily available data. To measure secretariat size, we use data on permanent staff provided by the Yearbook of International Organizations for the last reported year and include the logged number of staff (*staffsize*) to deal with the skewed distribution. Where the Yearbook does not provide data, we have done additional online research on the websites of the organizations. To measure budget size (*budget*), we have collected data on the budgets of IOs in the fiscal year 2019–2020 from annual reports. Ideally, we would have included variables for the type of budgetary funding as well as the flexibility of budgetary rules during crisis situations. These are unfortunately not available for these 75 IOs. Indeed, even exact budget numbers are not available for 29 of the 75 IOs. We therefore decided to measure budget in categorical form, differentiating between IOs with small (<US$100 million), medium (100 million to 1 billion) and large budgets (>1 billion). We infer budget size for those IOs that do not provide exact numbers for 2019 from reporting in previous years or descriptions in secondary literature. As robustness check, we also included a variable using exact budget numbers (in US$million, logged), *budget_est*, with estimations for missing values based on the average budgets of all IOs in the reference category (Table D, Appendix). Finally, we include a variable measuring type of *leadership* to differentiate if the head of secretariat (Secretary General or Director General) has previous high‐level political experience (President/Prime Minister/Vice/Foreign Minister or Secretary General of another IO).

We include several control variables. To measure the relevance of COVID‐19 for the 75 different IOs, we include the aggregated number of *COVID‐19* cases (logged) of all IO member states by 31 March 2020 as compiled by Johns Hopkins University. We expect that IOs whose members were particularly badly affected by COVID‐19 at the start of the pandemic will feel more pressure to respond to the exogenous shock. In addition, we include a control variable *policy field* (Hooghe et al. 2017), to account for the fact that IOs dealing with health, economics, and finance, as well as border management and migration issues will be most affected in their work and therefore also more likely to respond. While COVID‐19 is a challenge for most IOs and cuts across policy fields, from education to postal delivery, it is nevertheless important to control for those policies that caused most challenges.

In addition to the differential impact of COVID‐19, we control for several additional design features. First, we control for *policy scope* of IOs. Hooghe et al. (2019) convincingly distinguish between task‐specific and general purpose IOs, arguing that the scope of general purpose IOs is likely to expand over time, while the scope of task‐specific IOs remains relatively fixed. A general purpose IO, such as the EU, might become active in health policy over time, whereas the International Criminal Court is unlikely to do so. Second, we control for the *number of member states* (logged) (Pevehouse et al., 2020) with the expectation that IOs with a large membership will have more difficulty expanding policies in light of COVID‐19. Third, we include a dichotomous variable *power politics* indicating whether both China and the United States are members of an IO. Since many commentators have pointed at American–Chinese rivalry, we would expect IOs that include both countries as members to be unable to develop ambitious policy responses.

## 5 Analysis and discussion

We use ordered logit models (McCullagh, 1980) with robust standard errors due to the ordinal measurement of our dependent variable. The analysis presented in Table 2 reports results for five models. Model (1) shows results for hypotheses 1a and 1b on the effects of pooling and delegation of authority on policy responses, model (2) uses the sub‐dimensions for pooling and delegation, and model (3) reports results for Hypotheses 2a, b, and c about the effect of bureaucratic capacity, namely size of staff, budget, and leadership. Model (4) is a fully saturated model and Model 5 tests an interaction effect between staff and authority.^3^ Additionally, we report results of these five models with a differently scaled dependent variable in the Appendix (Table C) to test if results hold with collapsed categories (low = 0 and 1, medium = 2 and 3, high = 4 and 5), thereby increasing the number of observations for each category. The results remain largely the same.

**Table 2:** Determinants of policy responses

	(1)	(2)	(3)	(4)	(5)
Delegation	1.334			–0.827	
	(1.632)			(2.257)	
Pooling	–0.765			–1.853	
	(2.100)			(2.120)	
**delpolicy**		**4.417^+^**			4.520
		(2.596)			(4.226)
delbudget		–2.601			
		(2.267)			
poolpolicy		–0.887			
		(1.053)			
poolbudget		0.469			
		(1.212)			
**Staff (log)**			0.495*	0.499*	0.975**
			**(0.211)**	**(0.209)**	**(0.308)**
Leadership			–0.0172	–0.226	
			(0.571)	(0.714)	
Budget					
Medium			0.873	0.992	
			(0.793)	(0.827)	
High			1.074	1.218	
			(1.027)	(1.064)	
staffsize#delpolicy					–0.845
					(0.737)
IO Members (log)	0.0633	0.0553	–0.367	–0.142	–0.469
	(0.394)	(0.341)	(0.371)	(0.463)	(0.360)
US–China	0.607	0.632	0.210	0.168	0.265
	(0.734)	(0.745)	(0.768)	(0.779)	(0.715)
**Policy Scope**	2.079**	2.111**	1.981*	2.187*	2.204*
	**(0.738)**	**(0.684)**	**(0.810)**	**(0.935)**	**(1.023)**
**COVID Cases (log)**	**0.346^+^**	**0.337^+^**	0.346	0.320	**0.442^+^**
	**(0.191)**	**(0.196)**	(0.223)	(0.226)	**(0.238)**
Policy Field	0.222	0.160	0.750	0.664	0.707
	(0.543)	(0.554)	(0.568)	(0.583)	(0.503)
cut1	3.183	3.244	4.319^+^	3.998	7.269*
	(1.949)	(2.259)	(2.536)	(2.579)	(3.250)
cut2	4.300*	4.417*	5.655*	5.352*	8.688**
	(1.920)	(2.237)	(2.557)	(2.571)	(3.355)
cut3	4.893*	5.031*	6.378*	6.090*	9.430**
	(1.921)	(2.242)	(2.579)	(2.588)	(3.398)
cut4	6.572***	6.773**	8.528**	8.283**	11.55**
	(1.973)	(2.281)	(2.684)	(2.686)	(3.538)
cut5	8.248***	8.484***	10.69***	10.45***	13.64***
	(1.969)	(2.246)	(2.803)	(2.790)	(3.488)
*N*	75	75	73	73	73

Notes

Standard errors in parentheses ^+^ p < 0.10, * p < 0.05, ** p < 0.01, *** p < 0.001; Coefficients reported. The labels/numbers in bold are the variables and data that are statistically significant.

**Figure 2:**
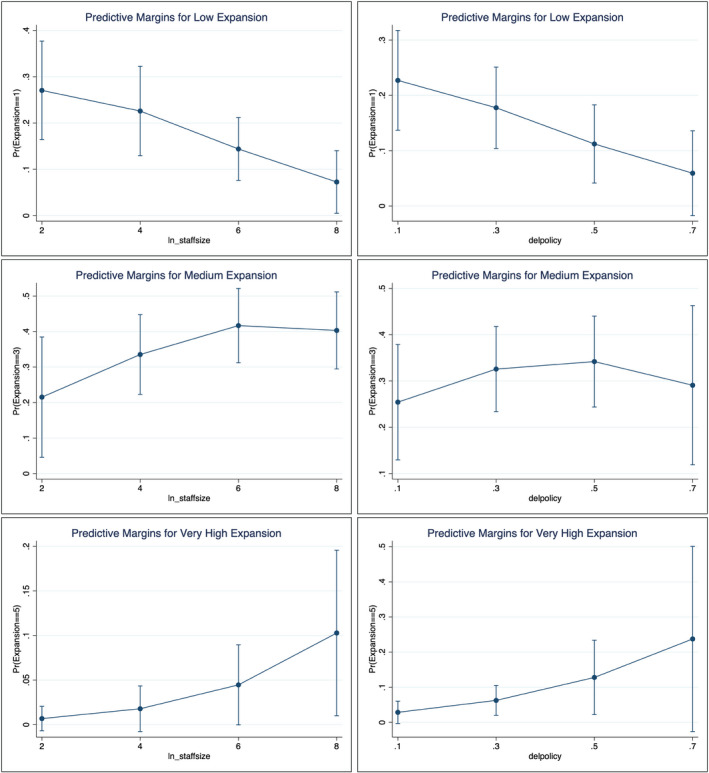
Predictive margins by size of staff (left) and delegated policy making authority (right) at low, medium, and very high levels of expansion in scope and policy instruments (with 90% confidence interval)

The models reveal interesting results. First, size of staff is one of the most important predictors to explain policy responses at the 0.05 significance level. In fact, an IO with ten times more staff has a 1.6 times higher probability of responding to the crisis in some way (dv = 1 to 5) instead of shutting down and doing nothing (dv = 0). The left‐hand side of Figure 2 explores the magnitude of this effect for relative probabilities that an IO exhibits low, medium, and very high levels of scope and policy instrument expansion. Substantively, they show that increasing staff size has a large effect on the probability that an IO has expanded its policy scope or instruments, with large IOs being highly unlikely to barely engage with COVID‐19 at all.^4^ We can thus accept Hypothesis 2a: IOs with a larger secretariat will more likely expand their scope and policy instruments during crises.

In contrast, neither budget nor leadership seniority are significant predictors, leading us to reject H2b and H2c. However, when including real budgetary data with estimates for missing values (Table D, Appendix), budget turns significant at the 0.1 significance level, pointing at the potential relevance of budget as a predictor of scope and instrument expansion. We also have to reject H1a and H1b on the effect of pooling and delegation. However, model (2) shows that this is not true for the more specific variable that measures power‐setting agenda with regard to policy making, which is significant at the 0.1 level. While we have to be careful with our conclusions at these significance levels, given the relatively small sample‐size we think it is reasonable to highlight these results. The right‐hand side of Figure 2 further exemplifies these findings: while the odds of being in the low category decrease with increasing policy agenda‐setting authority and are relatively stable for the medium category (continuity of functions), the odds increase for the very high category (exploit crisis as opportunity). This positive effect of delegated policy agenda‐setting power on the probability of scope and instrument expansion is even stronger if we collapse the categories of the dependent variable to three (see Table C, Model 2, Appendix).

Finally, model (5) tests to what extent larger bureaucracies with more delegated authority are more likely to expand their scope and policy instruments. Arguably, bureaucracies will only be able to move forward if they have at least some delegated authority to set the agenda and push for their proposals. However, the interaction effect itself is not significant, while the main effect of staff size remains highly significant. This could indicate that large bureaucracies are relevant during the COVID‐19 crisis even if they do not have the formal power of agenda‐setting. The interaction effect is significant in the alternative model with collapsed categories for the dependent variable (see Table C, Model 5, Appendix). Graphing the interaction reveals that the marginal effect of increasing policy agenda‐setting authority is only significant for very large bureaucracies (with staff above roughly 400). The marginal effect of policy‐agenda power on the probability of high scope and instrument expansion (dv = high) decreases with growing staff size, while it increases for the probability of being able to offer continuation of functions (dv = medium). Thus, model (5) in Table C confirms that IOs with larger bureaucracies might not necessarily need policy agenda‐setting authority to push for innovations, but that they may profit from it when it comes to keeping up shop during crisis.

These findings merit discussion. It is surprising that H1a and H1b on delegated and pooled sovereignty are not significant and this indirectly challenges the keynote work by Hooghe et al. (2019). As aggregate measures, however, these concepts include a number of sub‐dimensions that are not necessarily important in crisis situations with a short‐term time horizon. As noted above, formal authority is important as it determines where and how policy responses and decisions are made. At the same time, we also know that when the stakes are high, informal modes of governance may prevail (Stone, 2011) over the formal rules which are so central in the work of Hooghe et al. (2019). This may also help to explain the significance of secretariat staff. Ultimately, secretariats provide most continuity to IOs, as member states may not meet in the plenary and executive organs on a daily basis. In fact, the pandemic has even forced many IOs to postpone their regular annual plenary meetings, so many of the short‐term decisions were taken by IO bureaucrats or only had to pass through executive committees. As such, secretariats are the ones that need to deal with crises and if they have substantial expertise, they are even more likely to be leading in formulating policy responses.

That H2a is confirmed shows, once more, the importance of secretariat staff in the engine room of IOs. It also confirms bureaucratic theories that portray secretariat officials as opportunistic agents (Hawkins et al., 2006). At the same time, the adaptability of large bureaucracies is not a given (Barnett and Finnemore, 2004) and IO secretariats still need to work in tandem with key member states to actually achieve desired change in the long run (Dijkstra, 2017; Eckhard et al., 2019). These conflicting dynamics we have also seen empirically, for instance, in the case of the EU. The initial response by the European Commission to COVID‐19 was slow. A leading Member of the European Parliament wrote an op‐ed entitled ‘Avanti, von der Leyen!’ (Verhofstadt, 2020) and Commission President Von der Leyen even issued a ‘heartfelt apology’ to Italy for the slow EU response (BBC, 2020). Afterwards, however, the European Commission fully engaged, not just to tackle immediate health and economic challenges, but also to use COVID‐19 and the new recovery fund of 750bn euro to promote previous Commission priorities such as climate change, digitalization, and research.

H2b on budget and H2c on leadership were not confirmed. It is, however, important to stress the lack of detailed cross‐sectional data on bureaucratic capacity. While strong data now exist on pooling and delegation of sovereignty (Hooghe et al., 2017), the operationalization of budgets and leadership capacity (and even staff size) is more limited. In fact, results from robustness checks reported in Table D in the Appendix that use more fine‐grained data on IO budgets show that budgetary capacity could in fact be a significant predictor for the ability of IOs to profit from crisis. We know from scholarship on the resourcing of IOs that the idiosyncrasies of individual IOs matter beyond overall budget data: whether IOs can rely on core funding in the regular budget or voluntary funding by donors, the extent to which funding is earmarked, and what budgetary flexibility is available (Goetz and Patz, 2017; Graham, 2015, 2017; Patz and Goetz, 2019). A similar caveat also applies for H2c on leadership. Our operationalization is limited, and it is not always a priori clear whether the previous background of heads of secretariats determines leadership abilities during crises.

Qualitative evidence provides further support for the potential relevance of these variables. The WTO (coded as medium‐low) experienced a leadership crisis with its Director‐General stepping down in the middle of the first wave. The United Nations Educational, Scientific and Cultural Organization (UNESCO) (also medium‐low) was marred by continuous budgetary restraints over recent years, which may have prevented it from the development of more innovative solutions for the heavily affected policy domain of education. These findings thus also further underline the need for cross‐sectional data gathering on the bureaucratic capacity of IOs.

The control variables furthermore deserve attention. The number of COVID‐19 cases and the policy scope of IOs are significant predictors of the probability of scope expansion during the pandemic. IOs that are active in regions with high initial numbers of cases were more likely to not only provide a continuity of functions, but also to come up with innovative instruments and take on new tasks. Likewise, general purpose IOs are also more likely to expand their scopes and instruments which fits with expectations that task‐specific IOs have more stable policy portfolios (Hooghe et al., 2019). Surprisingly, policy field is not a significant predictor, despite our expectation that IOs in policy fields such as health, border management, or trade, would be most heavily affected by the pandemic. The findings suggest that exogenous shocks can be a real window of opportunity for nearly *all* IOs to branch out and take on new tasks.

Finally, the finding that geopolitics between the United States and China, measured in terms of both countries being a member of an IO, is not significant is also worth discussing. First, we have used quite a crude indicator for geopolitical contestation. At the same time, we see considerable variation in some of the geopolitically contested IOs. While the WHO was heavily affected by geopolitics, it has remained clearly the focal point for global health. Another example is the UN Security Council, which only managed to declare COVID‐19 a threat to international peace and security on 1 July 2020, whereas much of the rest of the UN organization had responded quicker. It is, in this respect, also worth pointing out that many of the plenary and executive bodies do not continuously meet and China and the United States may not have had the opportunity to actually block the implementation of policy initiatives. Similarly, much of IO day‐to‐day policy actually takes place outside the realm of great power politics, and it is up to the bureaucratic staff of an institution to take the lead when it comes to emergency politics.

## 6 Conclusions

Many observers have noted that COVID‐19 is yet another nail in the coffin of the liberal international order. At the same time, this article has argued that cross‐border crises such as COVID‐19 can present IOs with windows of opportunity to expand their scope and develop new policy instruments. It indeed shows that there is variation between how IOs have responded to COVID‐19: 22 IOs have hardly engaged with COVID‐19, 35 provide some continuity of operations and use existing instruments, and 18 IOs have expanded their scope and policy instruments. We find that the bureaucratic capacity of IOs (staff in secretariat) is significant to explain this variation, and to a lesser extent also the delegation of agenda‐setting authority. Furthermore, as previously found, this article also shows that general purpose IOs can more easily expand their scope than task‐specific IOs (Hooghe et al., 2019). And there is also some evidence that a higher number of COVID‐19 cases among the membership triggers a stronger response.

Significantly, we find that bureaucracies might be able to push their agenda even when they are lacking the formal authority to do so. When IO bureaucrats manage to use crisis moments as windows of opportunities to expand their scope and instruments, plenary bodies might well follow suit later on to legalize these practices and thus equip the IO with more authority (Jones et al., 2016; Kreuder‐Sonnen, 2019). In this respect, it will also be important to study the initial timing of IO policy responses to COVID‐19. Academic literature has highlighted the role of timing in public policy‐making (e.g. Agné, 2016; Eckhard et al., 2019; Howlett and Goetz, 2014), but so far, we know little about the effects of timing of policy responses on trajectories of institutional change. This is all the more important considering potential first‐mover advantages.

The current COVID‐19 crisis presents a clear test case to take stock of the current state of liberal international order. Just as Drezner (2014) claimed that ‘the system worked’ during the previous economic and financial crisis, this article shows that the large majority of IOs manage to continue their operations and some even gained from COVID‐19 in terms of policy scope and instruments. The effectiveness of IO response to COVID‐19 can be debated, but in terms of institutional change some IOs may be moving up and not down. The fact that many IOs as bastions of multilateralism are still standing raises questions about whether we are indeed seeing a crisis *of* liberal international order or *within* the liberal international order (Eilstrup‐Sangiovanni and Hofmann, 2020). We need to wait for the longer‐term impact on COVID‐19, but if IOs show resilience during a cross‐border crisis for which they were developed in the first place, their future is not necessarily bleak.

## Supporting information

Appendix S1

## Notes

2 Due to the small number of IOs in each category, confidence intervals are relatively large in each case. However, cut‐points between levels show a significant difference between categories.

3 We exclude three IOs because they are dead (COMECON, EAC1, NAFTA). NAFTA has been renegotiated in 2018 with the new agreement only taking effect on 1 June 2020.

4 Checks for multicollinearity between independent variables reveal no significant correlations. A likelihood ratio and brant test confirm that the proportional odds assumption is not violated.
