# Supplementary material for: COVID‐19 and Policy Responses by International Organizations: Crisis of Liberal International Order or Window of Opportunity?
Source: Glob Policy. 2021 Jun 23;12(4):443–54. doi: 10.1111/1758-5899.12975 (PMC8441772; doi:10.1111/1758-5899.12975)

**Online Appendix**

This is the online appendix accompanying the article of Debre, M. J. & Dijkstra, H. (2021). Covid-19 and policy responses by international organizations: Crisis of liber-al international order or window of opportunity? *Global Policy*.

Table A: Variables included in Models 1 to 5

| **Variable** | **Operationalization** | **Measurement** | **Source** |
| --- | --- | --- | --- |
| *Dependent Variable* | | | |
| Expansion | Change in Policy Scope and Policy Instruments  [no response (0); low (1) = discursive response;  low-medium (2) = either established task or policy instrument; medium (3) = both established tasks and policy instruments; high (4) = new task or new policy instrument; very high (5) = both new tasks and new policy instruments] | Ordinal | Own coding |
| *Independent Variables* | | | |
| Delegation | Degree of authority conferred to IO bodies | Continuous | Hooghe et al. (2017) |
| Pooling | Degree of joint decision-making in collective body | Continuous | Hooghe et al. (2017) |
| Bureaucratic Capacity |  |  |  |
| Staff | Number of permanent staff (logged) | Continuous | Yearbook of International Organizations |
| Budget | 2019 budget per number of member states | Continuous | Own coding |
| Leadership | Political experience in high executive position domestically or in IO | Dichotomous | Own coding |
| *Control Variables* | | | |
| Power Politics | US and China members | Dichotomous | Own coding |
| Size | Number of member states (logged) | Continuous | Pevehouse et al. (2020) |
| Covid-19 | Aggregated number of confirmed Covid-19 cases of all IO member states (logged) | Continuous | John Hopkins University |
| Policy Field | IO with mandate in health, finance/economics, or border control/migration | Dichotomous | Hooghe et al. (2017) |
| Policy Scope | Task-specific vs general purpose IO | Dichotomous | Hooghe et al. (2017) |

Table B: Values for policy responses and staff size for all IOs

| **IO Name** |  | **Policy Response** | **Size of Staff** |
| --- | --- | --- | --- |
| ACCT | Francophonie | Low-medium (2) | 200 |
| AMU | Arab Maghreb Union | Low-medium (2) | 50 |
| APEC | Asia-Pacific Economic Cooperation | Low (1) | 43 |
| ASEAN | Association of Southeast Asian Nations | Medium (3) | 50 |
| AU | African Union | High (4) | 1668 |
| Andean | Andean Community | Medium (3) | 150 |
| BENELUX | Benelux Union | Low-medium (2) | 49 |
| BIS | Bank for International Settlements | Low-medium (2) | 650 |
| CABI | Centre for Agriculture and Bioscience International | Low-medium (2) | 400 |
| CARICOM | Caribbean Community | High (4) | 238 |
| CCNR | Central Commission for the Navigation of the Rhine | Medium (3) | 28 |
| CEMAC | Central African Economic and Monetary Union | Low (1) | 130 |
| CERN | European Organization for Nuclear Research | Medium (3) | 2560 |
| CIS | Commonwealth of Independent States | Low (1) | . |
| COE | Council of Europe | Very High (5) | 2200 |
| COMESA | Common Market for Eastern and Southern Africa | Low (1) | 200 |
| ComSec | Commonwealth of Nations | Medium (3) | 380 |
| EAC2 | East African Community | High (4) | 361 |
| ECCAS | Economic Community of Central African States | Medium (3) | 100 |
| ECOWAS | Economic Community of West African States | Medium (3) | 260 |
| EEA | European Economic Area | Medium (3) | 14 |
| EFTA | European Free Trade Association | Medium (3) | 60 |
| ESA | European Space Agency | Medium (3) | 2043 |
| EU | European Union | Very High (5) | 32000 |
| EURAMET | European Association of National Metrology Institutes | No response (0) | 38 |
| FAO | Food and Agriculture Organization | Medium (3) | 3248 |
| GCC | Gulf Cooperation Council | No response (0) | . |
| GEF | Global Environment Facility | Low-medium (2) | 78 |
| IAEA | International Atomic Energy Agency | Medium (3) | 2200 |
| IBRD | World Bank | High (4) | 6800 |
| ICAO | International Civil Aviation Organization | Medium (3) | 700 |
| ICC | International Criminal Court | Low (1) | 858 |
| ICO | International Coffee Organization | Low (1) | 13 |
| IGAD | Intergovernmental Authority on Development | Medium (3) | 430 |
| ILO | International Labour Organization | Medium (3) | 2700 |
| IMF | International Monetary Fund | High (4) | 2400 |
| IMO | International Maritime Organization | Medium (3) | 300 |
| INTERPOL | International Criminal Police Organization | High (4) | 850 |
| IOMig | International Organization for Migration | High (4) | 9000 |
| ISA | International Seabed Authority | No response (0) | 34 |
| ITU | International Telecommunication Union | Low-medium (2) | 700 |
| IWhale | International Whaling Commission | No response (0) | 17 |
| LAIA | Latin American Integration Association | No response (0) | 65 |
| LOAS | League of Arab States | Low (1) | 460 |
| Mercosur | Common Market of the South | Medium (3) | 26 |
| NAFO | Northwest Atlantic Fisheries Organization | No response (0) | 11 |
| NATO | North Atlantic Treaty Organization | High (4) | 7500 |
| NordC | Nordic Council | Medium (3) | 15 |
| OAPEC | Organization of Arab Petroleum Exporting Countries | No response (0) | 53 |
| OAS | Organization of American States | High (4) | 750 |
| OECD | Organization for Economic Cooperation and Development | Medium (3) | 2500 |
| OECS | Organization of Eastern Caribbean States | Low (1) | 168 |
| OIC | Organization of Islamic Cooperation | Very High (5) | 200 |
| OPEC | Organization of the Petroleum Exporting Countries | High (4) | 139 |
| OSCE | Organization for Security and Cooperation in Europe | High (4) | 3462 |
| OTIF | Intergovernmental Organization for International Carriage by Rail | Low (1) | 20 |
| PCA | Permanent Court of Arbitration | No response (0) | 50 |
| PIF | Pacific Islands Forum | Medium (3) | 80 |
| SAARC | South Asian Association for Regional Cooperation | No response (0) | 61 |
| SACU | Southern African Customs Union | Low (1) | 374 |
| SADC | Southern African Development Community | Medium (3) | 92 |
| SCO | Shanghai Cooperation Organization | Low (1) | 60 |
| SELA | Latin American and Caribbean Economic System | No response (0) | 42 |
| SICA | Central American Integration System | Very High (5) | 50 |
| SPC | South Pacific Community | Medium (3) | 14 |
| UN | United Nations | High (4) | 38105 |
| UNESCO | UN Education, Scientific and Cultural Organization | Low-medium (2) | 2000 |
| UNIDO | UN Industrial Development Organization | Medium (3) | 670 |
| UNWTO | World Tourism Organization | Medium (3) | 110 |
| UPU | Universal Postal Union | Medium (3) | 250 |
| WCO | World Customs Organization | High (4) | 194 |
| WHO | World Health Organization | Very High (5) | 4000 |
| WIPO | World Intellectual Property Organization | Medium (3) | 1248 |
| WMO | World Meteorological Organization | Low (1) | 285 |
| WTO | World Trade Organization | Low-medium (2) | 664 |

Table C: Policy Responses (for expansion = low, medium, high)

|  | (1) | (2) | (3) | (4) | (5) |
| --- | --- | --- | --- | --- | --- |
| Delegation | 0.669 |  |  | -1.944 |  |
|  | (1.609) |  |  | (2.309) |  |
| Pooling | -0.825 |  |  | -2.079 |  |
|  | (2.106) |  |  | (2.222) |  |
| **delpolicy** |  | **5.168^*^** |  |  | **7.236^*^** |
|  |  | **(2.514)** |  |  | **(3.508)** |
| **delbudget** |  | **-4.103^+^** |  |  |  |
|  |  | **(2.197)** |  |  |  |
| poolpolicy |  | -0.310 |  |  |  |
|  |  | (1.126) |  |  |  |
| poolbudget |  | 0.197 |  |  |  |
|  |  | (1.145) |  |  |  |
| **Staff (log)** |  |  | **0.476^+^** | **0.513^*^** | **1.330^***^** |
|  |  |  | **(0.249)** | **(0.250)** | **(0.285)** |
| leadership |  |  | 0.143 | -0.115 |  |
|  |  |  | (0.628) | (0.721) |  |
| Budget |  |  |  |  |  |
|  |  |  |  |  |  |
| Medium |  |  | 1.210 | **1.407^+^** |  |
|  |  |  | (0.844) | **(0.802)** |  |
| High |  |  | 1.524 | 1.689 |  |
|  |  |  | (1.287) | (1.262) |  |
| **staff#delpolicy** |  |  |  |  | **-1.570^**^** |
|  |  |  |  |  | **(0.607)** |
| IO Members (log) | 0.0745 | 0.0369 | -0.430 | -0.181 | -0.643 |
|  | (0.420) | (0.366) | (0.452) | (0.545) | (0.452) |
| US-China | 0.996 | 1.101 | 0.822 | 0.801 | 0.973 |
|  | (0.765) | (0.763) | (0.748) | (0.753) | (0.703) |
| **Policy Scope** | **2.112^**^** | **2.201^**^** | **1.971^*^** | **2.230^*^** | **2.479^*^** |
|  | **(0.789)** | **(0.727)** | **(0.861)** | **(0.935)** | **(1.057)** |
| Covid Cases (log) | 0.353 | 0.355 | 0.348 | 0.311 | **0.513^+^** |
|  | (0.219) | (0.224) | (0.277) | (0.275) | **(0.307)** |
| Policy Field | 0.145 | 0.0411 | 0.655 | 0.612 | 0.590 |
|  | (0.531) | (0.535) | (0.577) | (0.600) | (0.536) |
| cut1 | 4.299^*^ | 4.306^*^ | 5.404^+^ | 4.989^+^ | 10.35^**^ |
|  | (2.037) | (2.132) | (2.874) | (2.769) | (3.503) |
|  |  |  |  |  |  |
| cut2 | 6.668^**^ | 6.843^**^ | 8.605^**^ | 8.239^**^ | 13.55^***^ |
|  | (2.169) | (2.262) | (3.136) | (3.044) | (3.821) |
| *N* | 75 | 75 | 73 | 73 | 73 |

Standard errors in parentheses ^+^ *p* < 0.10, ^*^ *p* < 0.05, ^**^ *p* < 0.01, ^***^ *p* < 0.001

Table D: Policy Responses (with exact budget data, missing values estimated)

|  | (1) | (2) | (3) | (4) | (5) |
| --- | --- | --- | --- | --- | --- |
| Delegation | 1.334 |  |  | -1.147 |  |
|  | (1.632) |  |  | (2.326) |  |
| Pooling | -0.765 |  |  | -2.234 |  |
|  | (2.100) |  |  | (2.136) |  |
| **delpolicy** |  | **4.417^+^** |  |  | 4.520 |
|  |  | **(2.596)** |  |  | (4.226) |
| delbudget |  | -2.601 |  |  |  |
|  |  | (2.267) |  |  |  |
| poolpolicy |  | -0.887 |  |  |  |
|  |  | (1.053) |  |  |  |
| poolbudget |  | 0.469 |  |  |  |
|  |  | (1.212) |  |  |  |
| **Staff (log)** |  |  | **0.361^+^** | **0.337^+^** | **0.975^**^** |
|  |  |  | **(0.211)** | **(0.200)** | **(0.308)** |
| leadership |  |  | 0.120 | -0.120 |  |
|  |  |  | (0.547) | (0.687) |  |
| **Budget_est (log)** |  |  | **0.341^+^** | **0.413^+^** |  |
|  |  |  | **(0.199)** | **(0.224)** |  |
| staffsize#delpolicy |  |  |  |  | -0.845 |
|  |  |  |  |  | (0.737) |
| IO members (log) | 0.0633 | 0.0553 | -0.407 | -0.135 | -0.469 |
|  | (0.394) | (0.341) | (0.359) | (0.449) | (0.360) |
| US-China | 0.607 | 0.632 | 0.399 | 0.387 | 0.265 |
|  | (0.734) | (0.745) | (0.753) | (0.753) | (0.715) |
| **Policy Scope** | **2.079^**^** | **2.111^**^** | **1.801^*^** | **2.023^*^** | **2.204^*^** |
|  | **(0.738)** | **(0.684)** | **(0.851)** | **(0.972)** | **(1.023)** |
| **Covid-19 (log)** | **0.346^+^** | **0.337^+^** | 0.318 | 0.274 | **0.442^+^** |
|  | **(0.191)** | **(0.196)** | (0.218) | (0.227) | **(0.238)** |
| Policy Field | 0.222 | 0.160 | 0.675 | 0.555 | 0.707 |
|  | (0.543) | (0.554) | (0.502) | (0.512) | (0.503) |
| / |  |  |  |  |  |
| cut1 | 3.183 | 3.244 | 4.065^+^ | 3.572 | 7.269^*^ |
|  | (1.949) | (2.259) | (2.368) | (2.440) | (3.250) |
|  |  |  |  |  |  |
| cut2 | 4.300^*^ | 4.417^*^ | 5.467^*^ | 5.008^*^ | 8.688^**^ |
|  | (1.920) | (2.237) | (2.384) | (2.422) | (3.355) |
|  |  |  |  |  |  |
| cut3 | 4.893^*^ | 5.031^*^ | 6.219^**^ | 5.786^*^ | 9.430^**^ |
|  | (1.921) | (2.242) | (2.402) | (2.432) | (3.398) |
|  |  |  |  |  |  |
| cut4 | 6.572^***^ | 6.773^**^ | 8.348^***^ | 7.971^**^ | 11.55^**^ |
|  | (1.973) | (2.281) | (2.531) | (2.562) | (3.538) |
|  |  |  |  |  |  |
| cut5 | 8.248^***^ | 8.484^***^ | 10.49^***^ | 10.12^***^ | 13.64^***^ |
|  | (1.969) | (2.246) | (2.627) | (2.628) | (3.488) |
| *N* | 75 | 75 | 73 | 73 | 73 |

Standard errors in parentheses

^+^ *p* < 0.10, ^*^ *p* < 0.05, ^**^ *p* < 0.01, ^***^ *p* < 0.001

Figure A: Interaction Effect


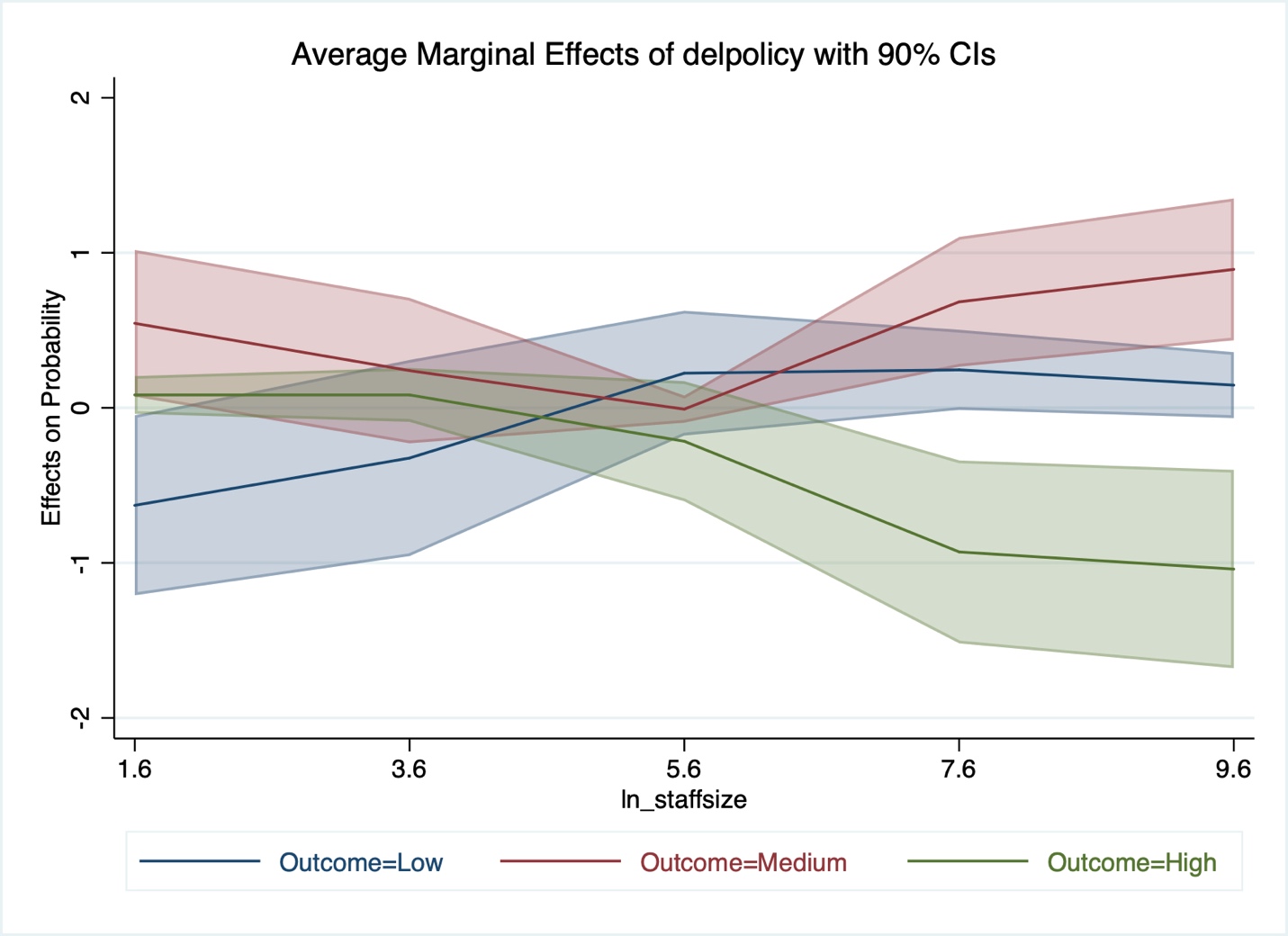

Supplement: Supplementary file 1 — Appendix S1 [file GPOL-12-443-s001.docx]
